# RESILIENCE AND AGE MODIFY INFLUENCES OF STRESS AND POSITIVE EXPERIENCES ON SUBJECTIVE COGNITION

**DOI:** 10.1093/geroni/igad104.2627

**Published:** 2023-12-21

**Authors:** Dakota Witzel, Suzanne Segerstrom, Maria Kurth, Soyoung Choun, Paris Crosby, Carolyn Aldwin

**Affiliations:** Penn State University, State College, Pennsylvania, United States; Oregon State University, Corvallis, Oregon, United States; Penn State University, University Park, Pennsylvania, United States; Oregon State University, Corvallis, Oregon, United States; University of Kentucky, Lexington, Kentucky, United States; Oregon State University, Corvallis, Oregon, United States

## Abstract

The COVID-19 pandemic is a major, chronic, worldwide stressor related to poorer health and well-being, especially among older adults, but less is known about risk and protective factors related to cognitive functioning during this stressful time. This eight-week study of 229 community-dwelling middle-aged and older adults (Mage=71.1; 74% women) examined associations between the intensities of weekly stress and positive experiences on subjective cognitive functioning. We also examined how age and trait resilience modified these associations. Zero-inflated Poisson models revealed a positive association between the intensity of stress and self-reported cognitive difficulties (γ=0.34, SE=0.07), whereas the intensity of positive experiences was associated with fewer difficulties (γ=-0.09, SE=0.03). Age moderated the effect of stress (γ=-0.01, SE=0.002), with the old-old having more subjective cognitive difficulties under more intense stress than the young-old. Resilience modified effects of stress and positive experiences, respectively, on cognition (γ=0.04, SE=0.01 and γ=-0.01, SE=0.01). Those with lower resilience reported more cognitive difficulties under more intense compared to less intense stress; this association was slightly attenuated for people with more resilience. Similar associations emerged between resilience and positive experience intensity. Older age was a risk factor for subjective cognitive difficulties during the COVID-19 pandemic, especially under conditions of high weekly stress, whereas resilience was a protective factor. Although those high in resilience appeared to be more sensitive to cognitive difficulties during more intense positive experiences, they still reported fewer difficulties than those low in resilience. Older adults’ cognitive resilience during the COVID-19 pandemic was heterogeneous and multifactorial.

